# Role of coagulation irregularities in cholangiocarcinoma: A review

**DOI:** 10.5937/jomb0-56538

**Published:** 2026-02-27

**Authors:** Hafsa Hamid, Sladjana Coric, Saira Rafaqa, Sanja Gluscevic, Filiz Mercantepe, Aleksandra Klisic

**Affiliations:** 1 Lahore College for Women University, Department of Biotechnology, Lahore, Pakistan; 2 Clinical Centre of Montenegro, Department of Cardiology, Podgorica, Montenegro; 3 Lahore College for Women University, Department of Zoology (Molecular Physiology and Endocrinology), Lahore, Punjab, Pakistan; 4 Clinical Centre of Montenegro, Department of Neurology, Podgorica, Montenegro; 5 Recep Tayyip Erdogan University, Faculty of Medicine, Department of Endocrinology and Metabolism, Rize, Turkey; 6 University of Montenegro, Faculty of Medicine, Podgorica, Montenegro; 7 Primary Health Care Centre, Centre for Laboratory Diagnostics, Podgorica, Montenegro

**Keywords:** cholangiocarcinoma, coagulation markers, pathogenesis, prognosis, holangiokarcinom, markeri koagulacije, patogeneza, prognoza

## Abstract

Cholangiocarcinoma (CCA) is a malignancy that originates from the biliary epithelium, presenting either intrahepatically or extrahepatically. A late-stage diagnosis and a poor overall prognosis characterise it. D-dimer, a biomarker indicative of coagulation and fibrinolysis activation, is believed to play a significant role in cancer progression, with elevated plasma levels correlating positively with metastatic disease and advanced cancer stages. In intrahepatic CCA, fibrinogen has been identified as an independent prognostic factor associated with adverse outcomes and reflects systemic inflammatory responses. The increased expression of urokinase-type plasminogen activator has been linked to lymphatic invasion and metastatic spread in CCA patients. Additionally, a preoperative elevation in prothrombin time may predict reduced survival rates for individuals undergoing potentially curative surgical interventions. P-selectin, which interacts predominantly with leukocyte ligands, has the potential to promote tumourigenesis and facilitate metastatic dissemination of mucin-producing carcinomas. Moreover, prolonged activated partial thromboplastin time may provide valuable insights into the extent of parenchymal involvement in CCA. This review aims to elucidate the role of coagulation abnormalities within the pathophysiology of cholangiocarcinoma.

## Abstract (sr)

Holangiokarcinom (HKA) je malignitet koji potiče iz biliarnog epitela i može da se javi intrahepatično ili ekstrahepatično. Karakterišu ga kasna dijagnoza i loša prognoza. Veruje se da D-dimer, biomarker koji ukazuje na aktivaciju koagulacije i fibrinolize, ima značajnu ulogu u progresiji karcinoma, pri čemu povišeni nivoi u plazmi pozitivno koreliraju sa metastazama i uznapredovalim stadijumima bolesti. Kod intrahepatičnog HKA, fibrinogen je identifikovan kao nezavisni prognostički faktor, povezan sa nepovoljnim ishodima i odrazom sistemskih inflamatornih odgovora. Povećana ekspresija urokinaznog tipa aktivatora plazminogena povezana je sa limfnom invazijom i meta statskim širenjem kod pacijenata sa HKA. Pored toga, preoperativno produženo protrombinsko vreme može predvideti smanjenu stopu pre'ivljavanja kod osoba koje se podvrgavaju potencijalno kurativnim hirurškim intervencijama. P-selektin, koji prete'no deluje na leukocitne ligande, ima potencijal da podstakne tumorogenezu i olakša meta statsku diseminaciju karcinoma koji proizvode mucin. štaviše, produženo aktivirano parcijalno tromboplastinsko vreme može da pruži koristan uvid u obim parenhimskog učešća u HKA. Ovaj pregled ima za cilj da razjasni ulogu abnormalnosti koagulacije u patofiziologiji holangiokar cinoma.

## Introduction

Cholangiocarcinoma (CCA) is a form of malignancy affecting the biliary epithelium, which can manifest either intrahepatically or extrahepatically. It is distinguished by late-stage diagnosis and a dire prognosis. CCA accounts for approximately 10% to 15% of all hepatobiliary cancers, making it the second most common primary liver cancer. Within CCA subtypes, distal and intrahepatic variants represent 20%–30% and 5%–10%, respectively, while hepatic cholangiocarcinomas constitute the remaining 60%– 70%. The global incidence of CCA has shown an upward trend in recent years, particularly in Western countries [1]. The pathogenesis of CCA is closely associated with liver inflammation, biliary inflammation, and cholestasis. Despite its poor prognosis, surgical intervention remains the only potential curative approach for CCA patients, with a five-year overall survival rate of less than 35% [2].

CCA encompasses a heterogeneous group of neoplasms originating from bile duct epithelial cells and ranks as the second most prevalent malignant liver tumour [3]. Its incidence is increasing worldwide, with a reported rate of 2.1 cases per 100,000 individuals annually in Western nations, and it peaks in the Eastern regions, notably in northwest Thailand. Currently, CCA is classified based on the anatomical site of the tumour: it is divided into extrahepatic cholangiocarcinoma (eCCA), which includes perihilar cholangiocarcinoma (pCCA) and distal cholangiocarcinoma (dCCA), and intrahepatic cholangiocarcinoma (iCCA), which is characterised by tumours originating within the liver [4].

Prognostic factors following potential curative resection for iCCA are yet to be fully elucidated, particularly since coagulopathy has been linked to poor outcomes in CCA [5][6]. In one clinical evaluation, for instance, at least one coagulation parameter abnormality was identified in 22.6% of patients, with prothrombin time (PT) being the most prevalent abnormality (8.87%). Patients exhibiting coagulopathy experienced significantly lower one-year survival rates compared to those with normal coagulation parameters [7].

PT has been identified as a potential prognostic indicator across several cancers, including CCA [8][9]. The mean overall survival (OS) for patients with low PT (PT<12.3 seconds) was significantly greater compared to those with high PT (23.03 months vs. 14.38 months, respectively, p=0.02) [8]. Similarly, the mean relapse-free survival (RFS) was superior in the low PT group than in the high PT group (17.78 months vs. 8.30 months, respectively, p=0.05) [8]. Thus, a preoperative elevation in PT may serve as a reliable predictor of adverse outcomes for patients undergoing curative treatment for CCA.

### Role of coagulation markers in the pathogenesis of CCA

**Figure 1:**
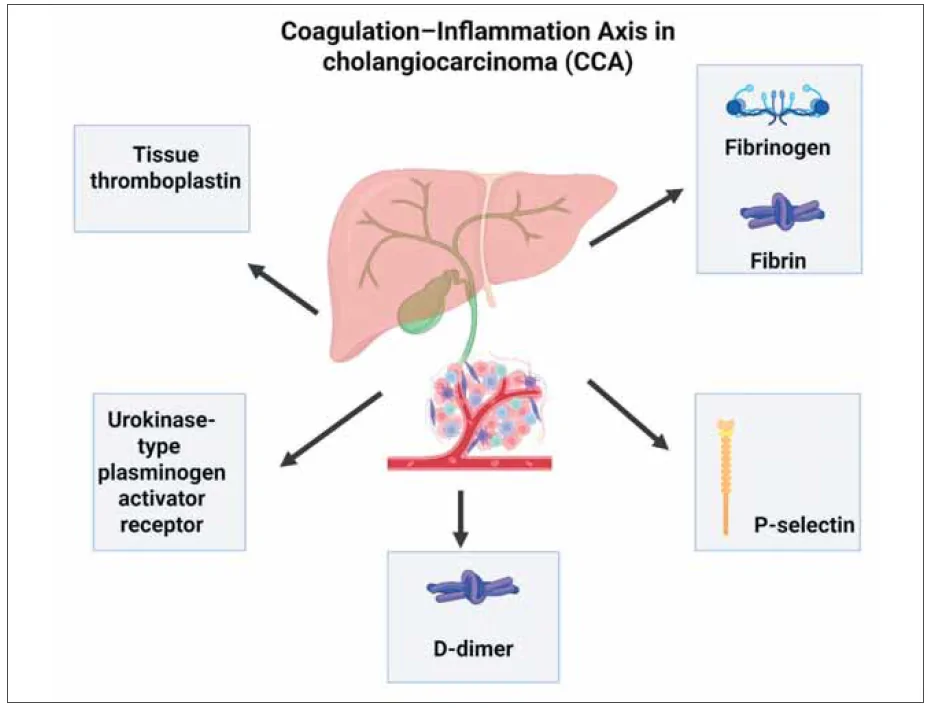
Pathophysiological roles of coagulation markers in cholangiocarcinoma (CCA).

This review focuses on selected coagulation markers – D-dimer, fibrinogen, plasminogen activator, prothrombin, P-selectin, and tissue thromboplastin – and their contributions to the pathogenesis of CCA. An overview of their roles is presented in Table 1 and illustrated graphically in the accompanying Figure 1.

**Table 1:** Summary of coagulation-related biomarkers associated with cholangiocarcinoma: circulating levels, mechanisms, and prognostic relevance. Legend: (↑)-increased levels, (?)-no studies have been reported yet

Coagulation <br>marker	Circulating <br>levels	Role in pathogenesis	Prognostic / Clinical significance	References
D-dimer	↑	Reflects activation of coagulation <br>and fibrinolysis; associated <br>with metastatic burden	Elevated levels predict poor overall <br>and progression-free survival	Chen et al., 2020; <br>Dai et al., 2018
Fibrinogen	↑	Promotes tumour stroma formation <br>and angiogenesis; reflects <br>systemic inflammation	Independent prognostic factor for <br>adverse outcomes and systemic <br>inflammatory response	Zhang et al., 2017; <br>Liu et al., 2021
uPA / uPAR	↑	Facilitates ECM degradation, <br>invasion, and metastasis	High baseline uPAR predicts poor <br>survival in unresectable CCA	Grunnet et al., 2014
Prothrombin time	↑	Indicates coagulopathy; a marker <br>of hepatic synthetic dysfunction	High PT correlates with shorter <br>OS and RFS after surgery	Wang et al., 2019
P-selectin	↑	Mediates tumour–platelet <br>interaction and metastasis of <br>mucin-secreting cancers	Overexpression associated with <br>aggressive tumour behaviour	Su et al., 2006
Tissue <br>thromboplastin	?	Initiates the extrinsic coagulation <br>pathway and inflammation	May reflect parenchymal <br>involvement rather than <br>biliary obstruction	Wiwanitkit, 2004

Summary of recent clinical studies investigating coagulation abnormalities in cholangiocarcinoma is presented in Table 2.

**Table 2:** Summary of recent clinical studies investigating coagulation abnormalities in cholangiocarcinoma (2016–2025).

Study	Population	Marker(s) studied	Main findings	Clinical implication
Chen et al., 2020	152 iCCA patients	D-dimer, CA19-9	CPDC score predicted <br>LNM and OS	Useful for surgical planning
Zhang et al., 2017	98 iCCA patients	Fibrinogen, NLR, PLR	High fibrinogen predicted <br>poor OS	Reflects systemic <br>inflammation
Wang et al., 2019	87 CCA patients	PT	High PT → lower OS/RFS	Independent <br>prognostic marker
Grunnet et al., 2014	48 inoperable CCA	uPAR	High baseline uPAR <br>= poor survival	Possible therapeutic <br>target

Tissue thromboplastin released from damaged hepatocytes activates coagulation cascades and correlates with parenchymal involvement. Fibrinogen and fibrin deposition in the tumour stroma promote tumour growth and metastasis. Elevated fibrinogen also reflects systemic inflammation and predicts poor prognosis. High urokinase-type plasminogen activator receptor (uPAR) expression is linked to lymphatic invasion, while elevated plasma D-dimer indicates metastatic progression. Increased P-selectin expression facilitates tumour–platelet interaction and metastatic spread of mucin-producing carcinoma cells. Pathophysiological aspects of prothrombin time, tissue factor, Von Willebrand factor and -thromboglobulin are not reported yet.

### D-dimer

D-dimer serves as a biomarker indicative of hemostatic dysfunction, closely associated with intravascular thrombosis, and indirectly reflects fibrinolysis and fibrin turnover. Vascular thrombi undergo systematic breakdown via fibrinolysis, producing D-dimer, a soluble fragment of fibrin degradation. This characteristic designates D-dimer as a significant marker for coagulation and fibrinolysis activation across numerous clinical scenarios [10].

In patients with iCCA undergoing curative resection, the prognostic value of D-dimer, in conjunction with preoperative CA19-9, was evaluated for predicting lymph node metastasis (LNM). The CPDC score emerged as an independent predictor for both LNM and OS during multivariate analysis, displaying an area under the curve (AUC) of 0.722 (p<0.001) for LNM prediction, and an AUC of 0.756 (p<0.001) for survival prediction. Notably, the predictive capacity of the CPDC score surpassed that of D-dimer and CA19-9 [11].

Patients with iCCA exhibiting a CPDC score of 2 demonstrated significantly poorer overall survival (median OS: 8.00 months vs. 19.00 months vs. not reached, p<0.001) and markedly decreased progression- free survival (PFS) (PFS: 4 months vs. 11 months vs. 15 months, p<0.001) compared to those with CPDC scores of 1 or 0, as established through Kaplan-Meier curve analysis. Significant differences in OS were identified across all groups, except in the HBsAg (+) cohort. Among groups characterised by tumours 5 cm, the presence of cirrhosis, and HBsAg (−), notable disparities in OS and PFS were observed, underscoring the practical prognostic value of the preoperative CPDC score for LNM and OS prediction in iCCA patients. This score can also assist in determining the requisite extent of lymph node dissection and guide follow-up strategies, particularly for cases with radiologically negative metastatic lymph nodes [11].

The association of D-dimer with cancer development is believed to be substantial. In a retrospective study, the correlation between plasma D-dimer levels and the advancement of various malignancies was corroborated. Plasma D-dimer levels were significantly elevated in patients with breast, gastric, pancreatic, colorectal, and rectal cancer compared to healthy controls. Additionally, a positive correlation was found between plasma D-dimer levels and both clinical cancer stage (p<0.05) and metastasis (p<0.05). These findings suggest that plasma D-dimer levels may serve as a valuable diagnostic tool for predicting cancer progression and metastasis [12].

A novel prognostic marker, D-dimer platelet multiplication (PDM), was evaluated in a study by Watanabe et al. for predicting outcomes in CCA patients [13]. In the recurrence cohort, a trend toward increased platelet counts accompanied by significantly elevated D-dimer and PDM was observed. Optimal cutoff values were established at 1.3 μg/mL for D-dimer, 245×10^4^/μL for platelet counts, and 158.2×10^4^ μg/mL×mL for PDM. Notably, high levels of platelets (p=0.05), PDM (p=0.005), and D-dimer (p=0.04) were associated with poor recurrence-free survival, and elevated platelet and PDM levels correlated with diminished cancer-specific survival (p=0.01). In this study, a multivariate analysis indicated that PDM exhibited the strongest correlation with CCA prognosis (p=0.006), and it served as an independent predictor of recurrence [13].

Increased levels of D-dimer, platelets, and PDM were linked to reduced recurrence-free and cancerspecific survival, thereby establishing PDM as a promising prognostic indicator for recurrence and overall outcomes in CCA patients [13].

### Fibrinogen and fibrin

Fibrinogen plays a crucial role in modulating plasma viscosity and promoting erythrocyte aggregation. It is essential for platelet aggregation, representing the terminal phase in the coagulation cascade that leads to fibrin formation. It is expressed both constitutively and in response to acute-phase reactions [14][15].

Many cancer patients exhibiting adverse clinical outcomes transition from a benign, localised growth pattern to an invasive, metastatic phenotype [16]. In the extracellular matrix (ECM), fibrinogen, along with other adhesive glycoproteins, is deposited during wound healing, promoting the binding of growth factors and enhancing cellular adhesion, migration, and proliferation during angiogenesis and tumour expansion. Malignant transformation and tumour progression arise from dysregulated synthesis and deposition of ECM components, correlating with aberrant control of cellular proliferation. Fibrin deposition typically occurs in the stroma of most tumour types [17].

A study by Zhang et al. [18] assessed the prognostic significance of plasma fibrinogen levels in patients with iCCA. The study explored the relationships between plasma fibrinogen levels and various inflammatory markers, including the neutrophil-lymphocyte ratio (NLR), platelet-lymphocyte ratio (PLR), and lymphocyte-monocyte ratio (LMR), serving as indicators of systemic inflammatory response (SIR). Findings revealed that elevated plasma fibrinogen levels significantly correlated with reduced OS and unfavourable prognosis (6.7 months in the high fibrinogen levels group versus 12.4 months in the low fibrinogen group; p=0.002). Additionally, plasma fibrinogen levels displayed a negative correlation with LMR and positive correlations with both NLR and PLR. Thus, elevated fibrinogen levels in iCCA patients can be utilised as an independent predictor of poor prognosis linked to SIR [18].

Li et al. [19] showed that the preoperative neutrophil count, fibrinogen-to-lymphocyte ratio and fibrinogen- to-lymphocyte-neutrophil score could be reliable biomarkers for predicting the prognosis of resectable extrahepatic cholangiocarcinoma.

Tumourigenesis and metastatic spread are intricately connected to systemic inflammation and nutritional status. The effectiveness of the fibrinogen/ albumin ratio index (FARI) in predicting RFS among patients with iCCA undergoing hepatectomy was addressed in a study by Liu et al. [20]. The Kaplan- Meier method investigated the association between patients classified as FARI-high versus FARI-low, utilising results from univariate and multivariate analyses to construct a nomogram. Kaplan-Meier analysis revealed that FARI-high correlates significantly with reduced RFS (p<0.001). The optimal cutoff value for FARI was determined to be 0.084 based on the Youden index from the ROC curve analysis. The nomogram developed based on FARI exhibited a Cindex of 0.663 and an AIC of 3081.07, accurately predicting RFS for iCCA patients post-hepatectomy. Preoperative FARI levels in patients undergoing hepatectomy for iCCA serve as independent prognostic indicators for RFS, contributing to informed post-surgical management strategies [20].

Aggressive tumours lead to a simultaneous increase in fibrinogen levels (indicating blood vessel invasion) and a decrease in albumin synthesis (denoting hepatic impairment), hence increasing FARI. Zeng et al. [21]. showed that preoperative values of NLR and FARI are linked with the surgical prognosis of patients with hepatocellular carcinoma. Xu et al. [22]. showed FARI as a reliable non-invasive parameter for predicting the recurrence-free survival (RFS) in patients with combined hepatocellular CCA.

### Plasminogen activator

The plasminogen activator (PA) system is an extracellular proteolytic enzyme system intricately linked to various physiological disorders. Accu mu - lating evidence indicates that components of this system – including urokinase-type plasminogen activator (uPA), its receptor (uPAR), and plasminogen activator inhibitors-1 and -2 (PAI-1 and PAI-2) – play critical roles in the proliferation and dissemination of malignancies. The binding of uPA to uPAR initiates extracellular matrix degradation, facilitating the migration of tumour cells from primary sites to distant metastatic locations, further aided by the conversion of plasminogen to plasmin. Components of the PA system, given their altered expression in various malignancies, represent ideal targets for diagnosis, prognosis, and therapeutic intervention aimed at reducing cancerrelated morbidity and mortality [23].

Targeting uPAR in cancer therapies offers advantages due to its low expression in healthy tissues, contrasted with elevated levels in malignant tumours. The invasion and metastasis of malignant tumours are closely correlated with uPAR, which significantly influences apoptosis, cell proliferation, tumour angiogenesis, and extracellular matrix (ECM) degradation. Furthermore, uPAR is associated with multidrug resistance (MDR) in tumour cells, a pivotal determinant of tumour aggressiveness and prognosis. Consequently, various anti-tumour therapies targeting uPAR have been developed to diminish drug resistance and inhibit tumour progression and metastasis [24].

Elevated levels of both intact and cleaved uPAR in blood and tissue samples have consistently been linked to poor survival outcomes in diverse cancer types; however, the prognostic relevance of uPAR in cholangiocarcinoma remains largely unexplored.

study involving patients with unresectable cholangiocarcinoma evaluated whether baseline levels of uPAR and changes in uPAR levels following chemotherapy can predict survival. Findings indicated that baseline uPAR(I–III) + uPAR(II–III) served as a significant predictor of survival (HR=2.08, p< 0.001). This predictive capability was validated by applying the linear predictor derived from a training cohort to a testing cohort, indicating that uPAR(I–III) + uPAR(II–III) predicted overall survival (p=0.049). Notably, high levels of uPAR(I–III) and uPAR(II–III) following two cycles of chemotherapy correlated with poor survival (HR=1.79, p=0.023), although this correlation lacked significance in the test cohort (p=0.21).

Therefore, survival prediction for patients with inoperable cholangiocarcinoma can be made by assessing baseline levels of uPAR(I–III) + uPAR(II–III) [25].

Immunohistochemical assessments revealed that uPA was expressed in 75.3% of CCA tissues, with increased uPA expression correlating with lymphatic invasion and metastasis in affected patients. Analysis through plasminogen-gelatin zymography indicated that both CCA cell lines produced membrane-associated and secreted forms of uPA, except for the H69 line. Notably, the HuCCA-1 and KKU-M213 CCA cell lines exhibited markedly elevated levels of uPA and uPAR; however, the KKU-M213 line exhibited significantly lower invasiveness in vitro, attributed to high expression of PAI-1. The necessity of uPA for CCA cell invasiveness was confirmed by observed reductions in invasiveness via siRNA or a specific uPA inhibitor (B428). Thus, combined in vitro and in vivo findings underscore the crucial role of uPA in CCA invasion, indicating its association with lymphatic invasion and metastasis, as well as its significant contribution to in vitro invasion. Consequently, uPA may serve as a promising therapeutic target [26][27].

### Prothrombin

Prothrombin, also known as coagulation factor II, is a vital protein within the blood coagulation cascade. It undergoes enzymatic cleavage to form thrombin (factor IIa), the active agent that catalyses the conversion of fibrinogen into fibrin, facilitating clot formation [28].

Prothrombin time (PT) has demonstrated predictive value for survival across various cancer types, including CCA. Wang et al. [8] specifically assessed the prognostic significance of PT levels in CCA patients. Elevated PT levels emerged as strong predictors of OS (HR=1.799, p=0.021) and RFS (HR=1.871, p=0.01) in CCA patients, independent of other variables (age, tumour differentiation, and TNM stage). The mean OS for the low PT group (PT<12.3 s) was significantly longer at 23.03 months compared to 14.38 months in the high PT group (PT 12.3 s; p=0.02). Similarly, the mean RFS was greater at 17.78 months for the low PT group compared to 8.30 months in the high PT group (p=0.05). These results indicate that elevated PT levels correlate with shorter OS (p=0.03) and poorer RFS (p=0.01). Thus, a preoperative increase in PT may serve as a reliable predictor of adverse outcomes in patients with cholangiocarcinoma undergoing curative surgical intervention [8].

### P-selectin

P-selectin is a crucial glycoprotein that functions as a key mediator in blood cell interactions. It facilitates the adhesion of activated platelets and endothelial cells to leukocytes, such as neutrophils and monocytes. Synthesised by endothelial cells, P-selectin is stored in specialised granules known as Weibel- Palade bodies, with additional storage observed in platelet alpha granules. Its primary role includes binding to the leukocyte-specific ligand known as P-selectin glycoprotein ligand-1 (PSGL-1), which promotes the initial migration of leukocytes toward inflamed endothelium and helps recruit them to sites of inflammation. Furthermore, P-selectin stimulates monocytes to generate tissue factor, an essential component for initiating the extrinsic pathway of blood coagulation [29].

Selectins operate as adhesion receptors that predominantly recognise specific vascular mucin-type glycoproteins bearing the sialyl-Lewisx carbohydrate structure. Elevated expressions of sialyl-Lewisx and tumour-associated mucins have been independently correlated with poor clinical prognosis and metastasis in various epithelial carcinomas. During metastatic progression, interactions between leukocytes, platelets, and tumour emboli with the endothelium of distant organs facilitate tumour dissemination. It has been suggested that P-selectin may contribute to tumour proliferation and enhance the metastatic potential of mucin-secreting cancers due to its role in adhesive interactions [30].

CD24, a cell surface protein initially identified in haematological malignancies, is also expressed across various solid tumours. P-selectin acts as an adhesion receptor that can bind to CD24. The overexpression of CD24 enhances the proliferation potential of cancer cells, indicating that CD24 expression may serve as a novel prognostic marker for intrahepatic cholangiocarcinoma [31].

### Tissue thromboplastin

Tissue thromboplastin, also referred to as CD142, is a pivotal protein that initiates the blood coagulation cascade in response to vascular injury by binding to and activating factor VIIa, a plasma serine protease. As an integral component of hemostasis, tissue factor plays a significant role in pathological conditions associated with coagulation. It stimulates the coagulation process across various thrombotic disorders, coagulopathies arising from sepsis, and other forms of disseminated intravascular coagulation. Recent studies have elucidated additional roles for tissue factor beyond hemostasis, including its involvement in cellular signalling, inflammatory processes, vasculogenesis, tumour growth, and metastasis [32].

Patients diagnosed with CCA frequently exhibit abnormal activated partial thromboplastin time (APTT), similar to patients with other liver diseases. A study analysing the correlations between patient characteristics and APTT levels revealed that APTT was not significantly correlated with most patient variables. However, significant correlations (p<0.05) were noted with aspartate aminotransferase (AST) and alanine aminotransferase (ALT). The strong correlation of APTT with AST and ALT – as opposed to serum bilirubin or alkaline phosphatase – suggests that prolonged APTT may be more closely linked to hepatic parenchymal injury rather than biliary obstruction [33]. Consequently, an extended APTT may serve as an indicator of substantial parenchymal involvement in CC [33].

### Conclusion and future perspectives

Cholangiocarcinoma represents a malignancy of the bile duct, significantly influenced by abnormalities in the coagulation system. This review emphasises the essential role that coagulation biomarkers play in the pathophysiology of CCA. Notably, the implications of factors such as prothrombin time, tissue factor, von Willebrand factor, and -thromboglobulin remain inadequately documented in current literature.

Vigilant monitoring and management of coagulation irregularities are critical for patients diagnosed with CCA, as these strategies may necessitate anticoagulant administration, particularly for those presenting with symptomatic thromboembolic complications. The identification and understanding of these coagulation markers could refine clinical decision-making, enhance prognostic accuracy, and ultimately improve therapeutic outcomes for patients with cholangiocarcinoma. Future research should aim to further elucidate the roles of coagulation markers in cholangiocarcinoma, paving the way for potential biomarkers that could guide individualised treatment approaches and improve patient prognosis.

## Dodatak

### Conflict of interest statement

All the authors declare that they have no conflict of interest in this work.

## References

[1] Bergquist A, von Seth E (2015). Epidemiology of cholangiocarcinoma. Best Pract Res Clin Gastroenterol.

[2] Maithel S K, Gamblin T, Kamel I, Corona V C P, Thomas M, Pawlik T M (2013). Multidisciplinary approaches to intrahepatic cholangiocarcinoma. Cancer.

[3] Banales J M, Cardinale V, Carpino G, Marzioni M, Andersen J B, Invernizzi P, Lind G E, Folseraas T, Forbes S J, Fouassier L, Geier A, Calvisi D F, Mertens J C, Trauner M (2016). Cholangiocarcinoma: Current knowledge and future perspectives consensus statement from the European Network for the Study of Cholangiocarcinoma (ENS-CCA). Nat Rev Gastroenterol Hepatol.

[4] Bridgewater J, Galle P R, Khan S A, Llovet J M, Park J, Patel T, Pawlik T M, Gores G J (2014). Guidelines for the diagnosis and management of intrahepatic cholangiocarcinoma. J Hepatol.

[5] Yoh T, Hatano E, Nishio T, Seo S, Taura K, Yasuchika K, Okajima H, Kaido T, Uemoto S (2016). Significant Improvement in Outcomes of Patients with Intrahepatic Cholangiocarcinoma after Surgery. World J Surg.

[6] Ruzzenente A, Conci S, Valdegamberi A, Pedrazzani C, Guglielmi A (2015). Role of surgery in the treatment of intrahepatic cholangiocarcinoma. Eur Rev Med Pharmacol Sci.

[7] Wang H, Liu W, Tian M, Tang Z, Jiang X, Zhou P, Ding Z, Peng Y, Dai Z, Qiu S, Zhou J, Fan J, Shi Y (2017). Coagulopathy associated with poor prognosis in intrahepatic cholangiocarcinoma patients after curative resection. Biosci Trends.

[8] Wang H, Ge X, Li Q, Nie J, Miao L (2019). Clinical Significance of Prothrombin Time in Cholangiocarcinoma Patients with Surgeries. Can J Gastroenterol Hepatol.

[9] Ke X, Jin B, You W, Chen Y, Xu H, Zhao H, Lu X, Sang X, Zhong S, Yang H, Mao Y, Du S (2021). Comprehensive analysis of coagulation indices for predicting survival in patients with biliary tract cancer. BMC Cancer.

[10] Johnson E, Schell J C, Rodgers G M (2019). The D-dimer assay. Am J Hematol.

[11] Chen Q, Zheng Y, Zhao H, Cai J, Wang L, Zhao J, Bi X, Li Z, Huang Z, Zhang Y, Zhou J, Wu J (2020). The combination of preoperative D-dimer and CA19-9 predicts lymph node metastasis and survival in intrahepatic cholangiocarcinoma patients after curative resection. Ann Transl Med.

[12] Dai H, Zhou H, Sun Y, Xu Z, Wang S, Feng T, Zhang P (2018). D-dimer as a potential clinical marker for predicting metastasis and progression in cancer. Biomed Rep.

[13] Watanabe A, Araki K, Hirai K, Kubo N, Igarashi T, Tsukagoshi M, Ishii N, Hoshino K, Kuwano H, Shirabe K (2016). A Novel Clinical Factor, D-Dimer Platelet Multiplication, May Predict Postoperative Recurrence and Prognosis for Patients with Cholangiocarcinoma. Ann Surg Oncol.

[14] Reinhart W H (2003). Fibrinogen: Marker or mediator of vascular disease?. Vasc Med.

[15] Rafaqat S, Gluscevic S, Patoulias D, Sharif S, Klisic A (2024). The Association between Coagulation and Atrial Fibrillation. Biomedicines.

[16] Azam A, Klisic A, Mercantepe F, Faseeh H, Mercantepe T, Rafaqat S (2024). Role of Coagulation Factors in Hepatocellular Carcinoma: A Literature Review. Life (Basel).

[17] Simpson H P J, Rybarczyk B (2001). Tumors and Fibrinogen: The Role of Fibrinogen as an Extracellular Matrix Protein. Ann N Y Acad Sci.

[18] Zhang C, Wang H, Ning Z, Xu L, Zhuang L, et al (2017). Hyperfibrinogen is associated with the systemic inflammatory response and predicts poor prognosis in intrahepatic cholangiocarcinoma. Int J Clin Exp Med.

[19] Li S, Zhang X, Lou C, Gu Y, Zhao J (2022). Preoperative peripheral blood inflammatory markers especially the fibrinogen-to-lymphocyte ratio and novel FLR-N score predict the prognosis of patients with early-stage resectable extrahepatic cholangiocarcinoma. Front Oncol.

[20] Liu H, Qiu G, Hu F, Wu H (2021). Fibrinogen/albumin ratio index is an independent predictor of recurrence-free survival in patients with intrahepatic cholangiocarcinoma following surgical resection. World J Surg Oncol.

[21] Zeng L (2025). Combined impact of prognostic nutritional index, fibrinogen-to-albumin ratio, and neutrophil-to-lymphocyte ratio on surgical outcomes and prognosis in hepatocellular carcinoma. Am J Cancer Res.

[22] Xu J, Li S, Feng Y, Zhang J, Peng Y, Wang X, Wang H (2022). The Fibrinogen/Albumin Ratio Index as an Independent Prognostic Biomarker for Patients with Combined Hepatocellular Cholangiocarcinoma After Surgery. Cancer Manag Res.

[23] Mahmood N, Mihalcioiu C, Rabbani S A (2018). Multifaceted Role of the Urokinase-Type Plasminogen Activator (uPA) and Its Receptor (uPAR): Diagnostic, Prognostic, and Therapeutic Applications. Front Oncol.

[24] Zhai B, Tian H, Sun J, Zou J, Zhang X, Cheng J, Shi Y, Fan Y, Guo D (2022). Urokinase-type plasminogen activator receptor (uPAR) as a therapeutic target in cancer. J Transl Med.

[25] Grunnet M, Christensen I J, Lassen U, Jensen L H, Lydolph M, Lund I K, Thurison T, Høyer-Hansen G, Mau-Sørensen M (2014). Prognostic significance of circulating intact and cleaved forms of urokinase plasminogen activator receptor in inoperable chemotherapy treated cholangiocarcinoma patients. Clin Biochem.

[26] Thummarati P (2012). High level of urokinase plasminogen activator contributes to cholangiocarcinoma invasion and metastasis. World J Gastroenterol.

[27] Hu M D, Jia L H, Wang M L (2023). PLAU contributes to the development of cholangiocarcinoma via activating NF-κB signaling pathway. Cell Biol Int.

[28] Wolberg A S (2007). Thrombin generation and fibrin clot structure. Blood Rev.

[29] Golubeva M G (2022). Role of P-Selectin in the Development of Hemostasis Disorders in COVID-19. Biology Bulletin Reviews.

[30] Kim Y J, Borsig L, Varki N M, Varki A (1998). P-selectin deficiency attenuates tumor growth and metastasis. Proc Natl Acad Sci U S A.

[31] Su M, Hsu C, Kao H, Jeng Y (2006). CD24 expression is a prognostic factor in intrahepatic cholangiocarcinoma. Cancer Lett.

[32] Morrissey J H (2004). Tissue Factor: A Key Molecule in Hemostatic and Nonhemostatic Systems. Int J Hematol.

[33] Wiwanitkit V (2004). Activated Partial Thromboplastin Time Abnormality in Patients with Cholangiocarcinoma. Clin Appl Thromb Hemost.

